# IDENTIFYING THE TRAJECTORIES OF MULTIMORBIDITY AND THEIR CORRELATES AMONG KOREAN OLDER ADULTS

**DOI:** 10.1093/geroni/igz038.2565

**Published:** 2019-11-08

**Authors:** Sun Ah Lee, Hey Jung Jun, Susanna Joo, Hye Won Chai

**Affiliations:** 1 Yonsei University, Seoul, Korea, Republic of; 2 The Pennsylvania State University, University Park, Pennsylvania, United States

## Abstract

Multimorbidity, the co-existence of two or more chronic diseases, has become prevalent among the older population. This study focused on identifying different patterns of multimorbidity trajectories across older adulthood and examining their predictors. We used six waves of the Korean Longitudinal Study of Aging (KLoSA), a nationally representative longitudinal data collected every two years from 2006 to 2016. The sample was older adults aged 65 years and older in 2006 (N=1,668). Multimorbidity was measured as the self-reported number of medically-diagnosed chronic diseases, and Growth Mixture Modeling was used to examine multimorbidity trajectories. Identified patterns of multimorbidity trajectories were then used as outcome variables in multinomial logistic regression models. Independent variables were socio-demographic, psychological, health-related behavioral and interpersonal factors at baseline. At Wave1, 76% of the sample had no or one chronic disease and 24% had two or more. At Wave6, 49% had none or one and 51% had two or more. Results identified four patterns of multimorbidity trajectory: “maintaining-low” (59.1%; reference), “maintaining-high” (7.3%), “moderately increasing”(26.4%), and “rapidly increasing” (7.2%). In terms of the correlates of these patterns, female older adults and respondents with higher depressive symptoms were more likely to be in the “maintaining-high” group. In addition, respondents who had less frequent meetings with friends, neighbors or relatives were more likely to be in the “rapidly increasing” group. The findings suggest that there are distinct patterns of multimorbidity trajectories across older adulthood, and interventions focusing on depressive symptoms or social engagement may be useful in preventing the increase in multimorbidity.

